# The Dark Side of the pollen: BSA-seq identified genomic regions linked to male sterility in globe artichoke

**DOI:** 10.1186/s12870-024-05119-z

**Published:** 2024-05-17

**Authors:** Matteo Martina, Aldana Zayas, Ezio Portis, Giovanna Di Nardo, Maria Francesca Polli, Cinzia Comino, Gianfranco Gilardi, Eugenia Martin, Alberto Acquadro

**Affiliations:** 1 DISAFA, Plant Genetics and Breeding, University of Turin, Turin, Italy; 2 IICAR (Instituto de Investigaciones en Ciencias Agrarias de Rosario), CONICET, Campo Exp. J.F. Villarino, Zavalla, Santa Fe Argentina; 3 DBIOS, Department of Life Sciences and Systems Biology, University of Turin, Turin, Italy

**Keywords:** Globe artichoke, BSA-seq, Male sterility, CYP702A3, Pollen vitality

## Abstract

Globe artichoke (*Cynara cardunculus* var. *scolymus*; 2n = 2x = 34) is a food crop consumed for its immature flower heads. Traditionally, globe artichoke varietal types are vegetatively propagated. However, seed propagation makes it possible to treat the crop as annual, increasing field uniformity and reducing farmers costs, as well as pathogens diffusion. Despite globe artichoke’s significant agricultural value and the critical role of heterosis in the development of superior varieties, the production of hybrids remains challenging without a reliable system for large-scale industrial seed production. Male sterility (MS) presents a promising avenue for overcoming these challenges by simplifying the hybridization process and enabling cost-effective seed production. However, within the *Cynara* genus, genic male sterility has been linked to three recessive loci in globe artichoke, with no definitive genetic mechanism elucidated to date. A 250 offsprings F_2_ population, derived from a cross between a MS globe artichoke and a male fertile (MF) cultivated cardoon (*C. cardunculus* var. *altilis*) and fitting a monogenic segregation model (3:1), was analyzed through BSA-seq, aiming at the identification of genomic regions/genes affecting male sterility. Four QTL regions were identified on chromosomes 4, 12, and 14. By analyzing the sequence around the highest pick on chromosome 14, a cytochrome P450 (*CYP703A2*) was identified, carrying a deleterious substitution (R/Q) fixed in the male sterile parent. A single dCAPS marker was developed around this SNP, allowing the discrimination between MS and MF genotypes within the population, suitable for applications in plant breeding programs. A 3D model of the protein was generated by homology modeling, revealing that the mutated amino acid is part of a highly conserved motif crucial for protein folding.

**Supplementary Information:**

The online version contains supplementary material available at 10.1186/s12870-024-05119-z.

## Key message

Globe artichoke (*Cynara cardunculus* var. *scolymus* L.) is a mediterranean allogamous, mainly cultivated for human consumption. The establishment of superior varieties through heterosis has been applied in this species, but their production is complex, and the development of male sterile varieties has been of interest among plant scientists and breeders as a cost-effective solution for hybrid seeds production. Here, we report the use of the BSA-seq approach for the identification of a SNP strictly associated with male sterility in an F_2_ mapping population, we developed a single dCAPS marker allowing the discrimination between MS and MF genotypes within the population, and we discussed a potential role for a candidate gene (CYP702A3), involved in pollen grain vitality, present in the candidate region.

**Supplementary Information:**

The online version contains supplementary material available at 10.1186/s12870-024-05119-z.

## Introduction

Globe artichoke (*Cynara cardunculus* var. *scolymus* L.) is a highly heterozygous, allogamous species native to the Mediterranean region, traditionally cultivated for human consumption of its fresh, corned, or frozen immature heads, characterized by high nutraceutical value [1, 2]. *C. cardunculus* includes two further taxa: (i) var. *sylvestris*, the progenitor of both cultivated forms, namely wild cardoon [3, 4], and (ii) the var. *altilis*, the cultivated cardoon, grown for the production of fleshy stems [5, 6]. Worldwide, with almost 115,897 ha and 1,516,955 t per year, Italy is the main producer country, accounting for about 24% of the world production [7]. Traditionally, this crop is cultivated in the Mediterranean basin (almost 85% of the world production), but in the last decades the production has developed also in other regions, such as Argentina, Peru, China, and USA [7, 8]. Artichoke production is traditionally carried out through vegetative propagation [9], although sexual propagation is possible [10]. However, in recent years a considerable number of new globe artichoke seed-propagated cultivars, open pollinated or hybrids, have been developed and successfully introduced in the market [11].

Historically, the use of heterosis for the establishment of superior varieties has been applied through the development of hybrids, but their production is complex in the absence of a system that allows large-scale crosses for industrial seeds production. For this reason, the mechanism of male sterility has long been of interest among plant scientists and breeders as a cost-effective solution for hybrid seeds production [12]. Among *Cynara* genus, genic male sterility has been associated with three recessive loci in globe artichoke only, but no clear genetic mechanism has been proposed [13–15]. Recently, Zayas et al. [16] developed an F_2_ population fitting a monogenic segregation model for MS, which was analyzed by combining the sequence related amplified polymorphism (SRAP) technology and the bulk segregant analysis (BSA) approach.

BSA allows the identification of genetic regions associated with traits of interest by selecting contrasting individuals within a segregating population, bulking the identified genotypes, and analyzing their genetic differences [17, 18]. This technique has been extensively applied with traditional molecular markers, both in qualitative traits investigation and quantitative trait loci (QTLs) mapping. In the NGS-era, its power has been dramatically improved by the application of whole genome sequencing (WGS − [19]). This approach is known as bulked segregant analysis by deep sequencing (BSA-seq) and has been shown to be highly reliable for QTL mapping in many species [19]. Its main advantage is that it can quickly associate a specific locus with candidate genomic regions, which considerably reduces workload and time. Thanks to the availability of a reference genome for the globe artichoke, released and implemented starting from an inbred variety harboring only 10% of heterozygosity [20–22], parental genotypes can then be investigated, and a large number of single nucleotide polymorphism (SNPs) and some insertion/deletion (InDels) can be identified, potentially associated with genomic regions of interest for the focused trait. While the potential of male sterility (MS) in globe artichoke offers a promising pathway for the efficient production of F_1_ hybrids, our understanding of its genetic underpinnings remains rudimentary, and only a few associated loci have been identified in the *Cynara* genus, without a clear delineation of the underlying genetic pathways. This study aims to address this gap by leveraging an F_2_ population developed from a cross between a MS globe artichoke and a male fertile cultivated cardoon, analyzed through bulk segregant analysis sequencing (BSA-seq). Here we identified specific genomic regions and candidate genes responsible for male sterility, with a focus on a cytochrome P450 gene variant, CYP703A2, suspected to impair male fertility. Our results provide a better delineation of the genetic architecture of MS in globe artichoke, which will not only fill a significant knowledge gap but also facilitate the development of molecular markers for breeding MS varieties, ultimately advancing globe artichoke breeding programs.

## Materials and methods

**Fig. 1:**
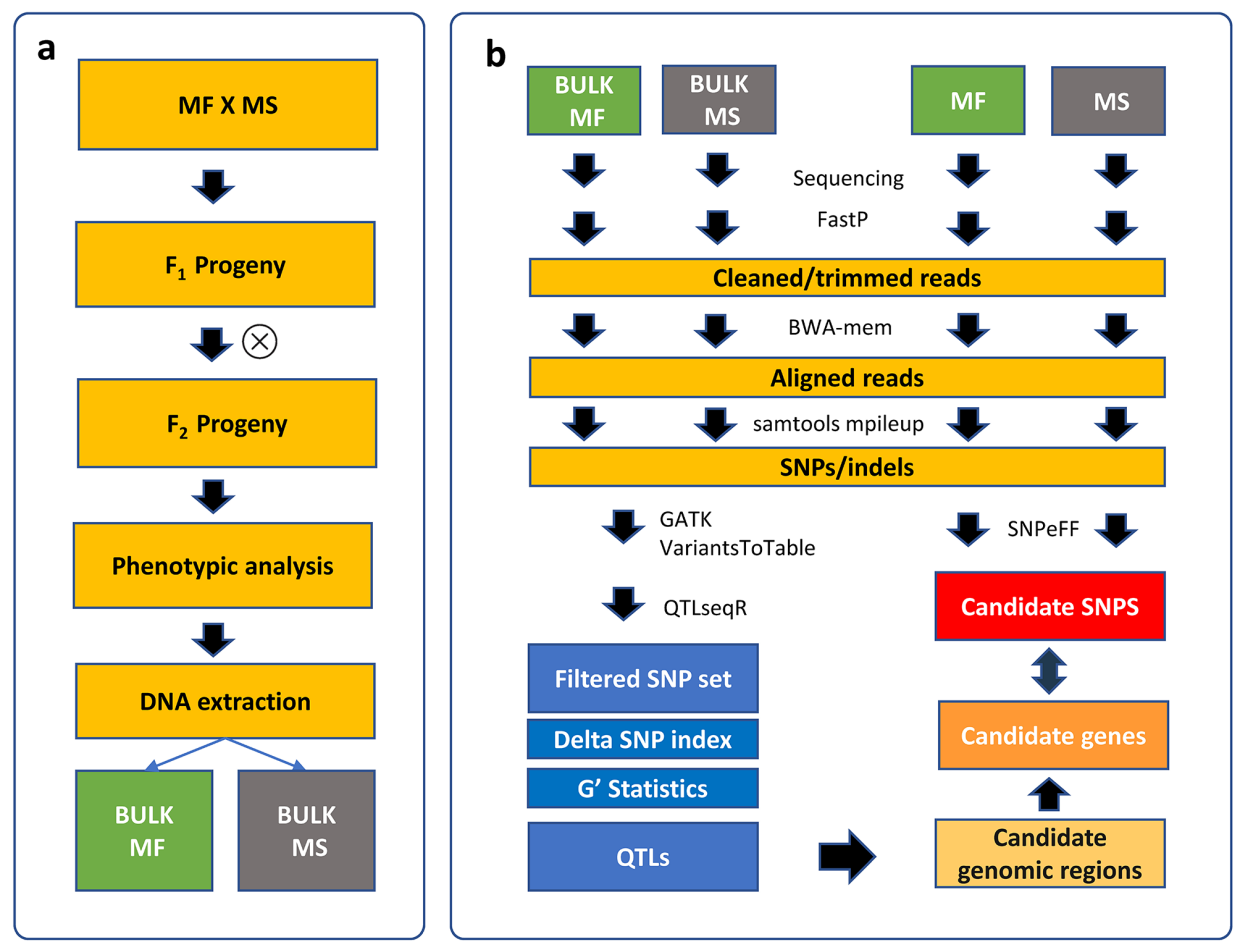
Overall presentation of the experimental design

Starting from the material developed by Zayas et al., 2019, BSAseq was applied for the identification of QTLs and potential candidate genes for male sterility in globe artichoke. A schematic representation of the experimental workflow is presented in Fig. 1.

### Plant material and bulks construction

An F_2_ population of 250 individuals was established by selfing an F_1_ individual obtained by crossing a male sterile globe artichoke genotype and a cultivated cardoon genotype and was grown as reported by Zayas et al. [16]. Pollen production was screened during two seasons, and two phenotypic groups were identified: male fertile (MF), plants with normal pollen production, and male sterile (MS), plants not producing pollen. Overall, 195 MF plants and 55 MS plants were phenotyped, fitting a 3:1 monogenic mendelian segregation model. Two bulks were constructed, assuming that the MS bulk (Bulk 1) included only homozygous recessive plants, and that the MF bulk (Bulk 2) included heterozygous and homozygous dominant individuals for the locus of interest.

### DNA extraction and bulk sequencing

Genomic DNA was extracted from fresh leaves of each parental genotype, as well as from 15 individuals of the MS group (Bulk 1) and 15 plants of the MF ones (Bulk 2), using DNeasy Plant mini Kit (QIAGEN). Nucleic acid quantification was performed with the Qubit 2.0 fluorometer (Qubit™ dsDNA BR Assay Kits, Thermo Fisher). Within every bulk, the fifteen genotypes were equimolarly pooled and used for library preparation. The pooled DNA samples were sequenced, together with the parental lines, at the Novogene UK sequencing facility using Illumina’s NovaSeq 6000, preparing standard Illumina sequencing libraries with 350 bp insert size. Whole-genome sequencing (PE150) was performed at 45x coverage on both the bulks and at 65x (MS) and 50x (MF) in the parental lines, obtaining an average of 33G raw data per sample.

### NGS-based BSA analysis

The raw reads obtained from the two bulks were cleaned and mapped on the globe artichoke reference genome (V2 - Acquadro et al. [22], http://www.artichokegenome.unito.it) using *FastP* [23] with standard filtering parameters, and Burrows-Wheeler Aligner program (BWA, v0.7.17, https://sourceforge.net/projects/bio-bwa/files) for the alignment. Variant calling was performed on the aligned sequences, identifying SNPs and INDELs between the sequenced bulks using Samtools mpileup, with minimum mapping quality equal to 25. SNPs having mapping quality lower than 20 were removed.

NGS-based BSA analysis was performed using the R package QTLseqr (https://github.com/bmansfeld/QTLseqr*)* developed by Mansfeld and Grumet [24] by calculating SNP index, deltaSNP index, and tricube-smoothed G value as described by Takagi et al. [25], Magwene et al. [18], and Yang et al. [26]. SNPs were filtered with standard parameters suggested by the pipeline, namely: refAlleleFreq = 0.20, minTotalDepth = 40, maxTotalDepth = 400, depthDifference = 100, minSampleDepth = 20, minGQ = 99. Based on the BSA-seq analysis, candidate regions surrounding QTL peaks were recorded. Those genomic coordinates were intersected with annotated SNP datasets.

### Progeny’s parents’ resequencing

Parental lines reads were cleaned using *FastP* [23] with standard filtering parameters and mapped onto globe artichoke genome reference (v2, Acquadro et al. [22]; http://www.artichokegenome.unito.it/.) using Burrows-Wheeler Aligner program (BWA, v0.7.17, https://sourceforge.net/projects/bio-bwa/files*).* Samtools mpileup was used for SNP calling, with minimum mapping quality equal to 25, and filtering SNPs call quality and depth. SNPs having mapping quality lower than 20 were removed. Common variants between parental lines were filtered out and SNPs were then analyzed using the SNPeff (http://pcingola.github.io/SnpEff/*)* suite to predict their effect on the set of gene models of globe artichoke. The effect of each SNP/indel was classified according to SNPeff software into four classes: (1) “modifier”; (2) “low” impact; (3) “moderate” impact and (4) “high” impact.

### SNP evaluation of the impact on the biological function

Genomic SNPs in parental lines datasets were analyzed, defining peak regions according to the slope rate around the main SNP in the region. In brief, considering that every significant SNP has to be evaluated as associated with the investigated trait in BSAseq analysis, candidate genes were hypothesized to be more densely located around sharper peaks than flat ones. Given the rapid increase and decrease of G’ around the top SNP in sharp peaks, a 500Kb genomic region was selected as candidate for them, while 800Kb was evaluated for flat peaks. Such intervals where further reduced to ∼ 100-200Kb, investigating the closest genes to the peak. In the peak regions, moderate/high impact mutations were considered and the ones in homozygous state were selected. Some candidate moderate impact SNPs were also submitted to Provean analysis (Protein Variation Effect Analyzer algorithm, https://www.jcvi.org/research/provean*)*, to check if the mutation had an impact on the biological protein functions. The score threshold used was set to the standard − 2.5 value.

### Homology modeling of CYP703A2

Homology models for both the WT and Arg424Gln CYP703A2 were built using the software Modeler 9.25 and the online tool SWISS-MODEL [27], using CYP76AH1 crystal structure from *Salvia miltiorrhiza* Bunge (PDB ID 5YLW) as a template. Energy minimization was performed using YASARA Amberff14SB force field and subjected to validation using Molprobity [28] and QMEAN [29]. The best models were selected according to their QMEAN4 score and to the percentage of residues in favored regions. The WT protein showed a QMEAN4 value of − 1.34, and the Ramachandran plot showed that 95.79% were in favored regions. The QMEAN4 value for Arg424Gln protein was − 1.69 and 96.41% of the residues were in the favored regions of the Ramachandran plot. The difference between the two models in the R424 region were analyzed in the UCSF Chimera software [30], which also allowed the calculation of the hydrogen bonds of the amino acid of interest.

### dCAPS primer design and experimental validation of DNA polymorphisms

To verify the MS mutation highlighted by Illumina alignment, Sanger sequencing was performed on a Applied Biosystems 3500 Series Genetic Analyzer using the BrilliantDie™ Terminator Cycle Sequencing kit by NimaGen according to standard protocols, followed by dCAPS marker development (http://helix.wustl.edu/dcaps/) using *HpaII* restriction enzyme. 1 µl of the genomic DNA was used as template in a 20 µl PCR containing 10 pmol of forward (GGACACTTTCTCTTTCCTGCA) and reverse primer (TGATATGGGATGATATCAACGTG), 1.5 mM MgCl_2_, 1 mM dNTP and 1 U Taq polymerase (GoTaq® DNA Polymerase) in the manufacturer’s buffer. The amplification program was 94°C/120’’, 25 cycles of 94 °C/30’’, 55 °C/30’’, 72 °C/60’’, and 10’ incubation at 72 °C. PCR products were digested by HpaII restriction endonuclease according to the producer’s instructions and run in a 1.5% agarose gel-based electrophoresis to verify polymorphisms and segregation pattern of the dCAPs marker.

## Results

To identify and locate the genomic regions and genes responsible for male sterility in globe artichoke, as well as to develop molecular markers to be applied in breeding programs, BSA-seq approach was applied in an F_2_ segregating population, previously developed and phenotyped for MS by Zayas et al. [16].

### Whole-genome sequencing of male sterile (MS) and male fertile (MF) bulks

We performed Illumina sequencing (45X coverage; 2 × 150 bp) of two bulks (MS bulk, and MF bulk), each one containing 15 genotypes concordant for the investigated trait, as well as the two parental lines (65X and 50X coverage for MS and MF, respectively). Genome sequencing of the two bulks yielded 447 million raw pair-end reads (in 224 million clusters), while the two parents yielded ∼ 569 million raw pair-end reads (in 284 million clusters, Table 1).

**Table 1:** Sequencing statistics

DNA	clusters	*N*° raw reads	bp	clusters	*N*° clean reads	bp	% cleaned	Final Coverage (X)	phenotype
MS parent	122,236,815	244,473,630	36,671,044,500	122,035,424	244,070,848	36,430,808,266	99.84%	50.2	sterile
MF parent	162,159,276	324,318,552	48,647,782,800	161,470,716	322,941,432	48,117,090,189	99.58%	66.3	fertile
Bulk male sterile	113,565,092	227,130,184	34,069,527,600	113,097,153	226,194,306	33,677,359,194	99.59%	46.4	sterile
Bulk male fertile	110,141,514	220,283,028	33,042,454,200	109,811,254	219,622,508	32,701,145,992	99.70%	45.0	fertile

The sequence data have been deposited into NCBI as Short Read Archive (SRA) files under the Bioproject PRJNA892759.

The sequenced reads of the two bulks were aligned to the reference genome (v2, Acquadro et al. [16]), detecting a total of 7,023,150 SNP/Indel variants. The two parents showed 8,203,723 and 4,748,963 SNPs/indels for MS and MF, respectively. A SnpEff analysis focused on coding regions (CDS) was also conducted on the MS parent. A total of 202,645 SNPs/indel in CDS were found (∼ 2.5% of the total genomic variants). The majority (∼ 51.2%) were non-synonymous (missense), followed by synonymous (silent; ∼47.6%), and nonsense (∼ 1.2%) mutations.

### BSA-seq analysis

To analyze the differences between contrasting phenotypes, two statistics are calculated (ΔSNP and G’), based on allele counts. The first statistic, ΔSNP, is calculated using the SNP index of the two bulks, which is determined by dividing the alternate allele depth by the total read depth. ΔSNP is the difference between the SNP index of the bulk defined as high and the bulk defined as low. The second statistic, G’, is a tricube-smoothed G statistic. Based on the alleles count, BSAseq can quickly associate a specific locus with candidate genomic regions.

**Fig. 2:**
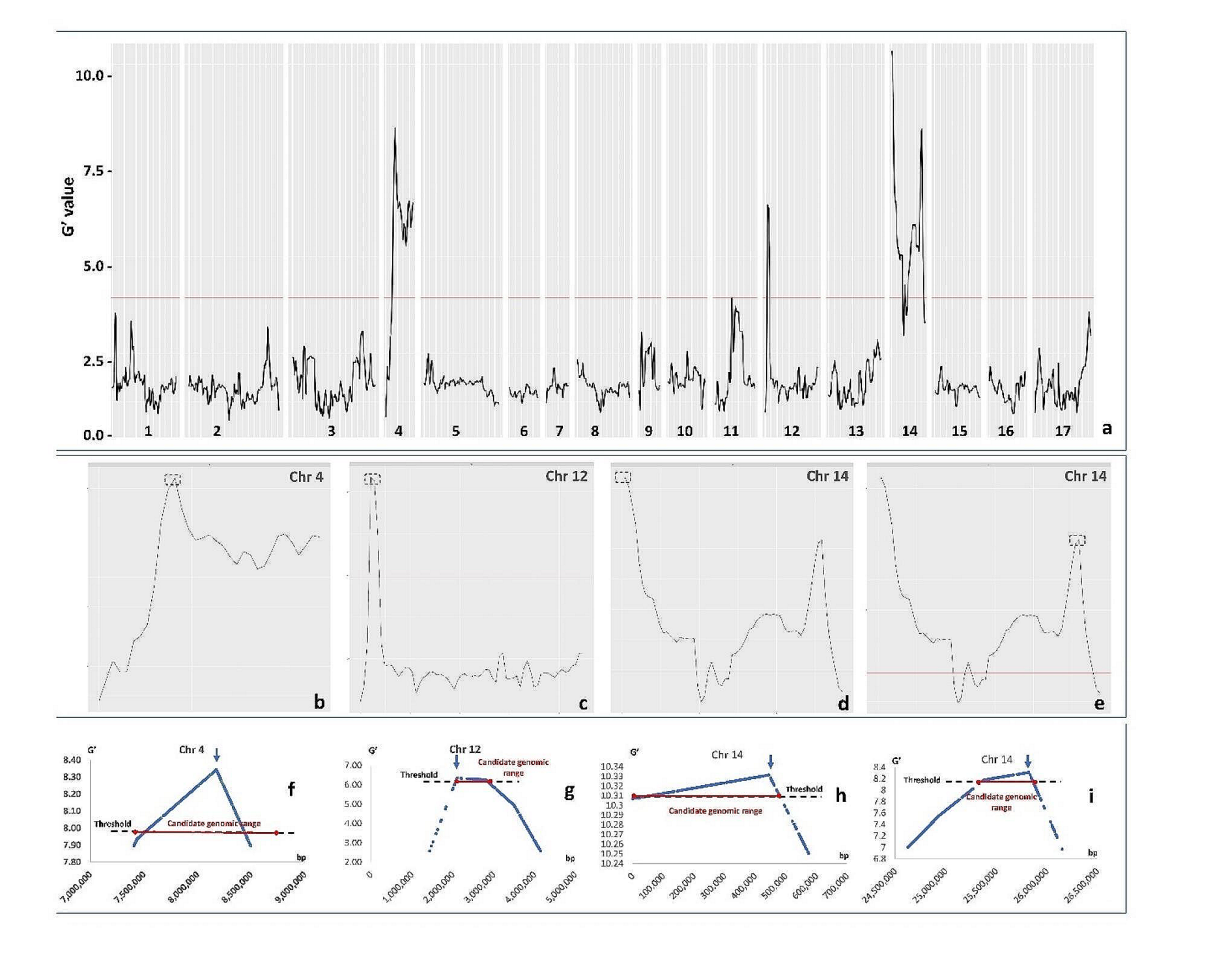
Quantitative trait loci (QTL) for male sterility identified by QTLseqr. Plots produced by the plotQTLStats() function with a 1 Mb sliding window: **a**) The tricube-smoothed G’ value. **b**-**e**) Detailed plots of the tricube-smoothed G’ value produced over chromosomes 4, 12 and 14; **f**-**i**) details of the peak regions and selected thresholds

BSA-seq analysis revealed four chromosomal regions (one on chromosome 4 and 12; two on chromosome 14) putatively involved in male sterility, showing G’ values above the threshold (G’ > 6.5; Fig. 2a, Suppl. Table 1).

All the regions surrounding the G’ peaks were investigated in the parental genomes showing many genes and SNPs. Sub-regions at the top of the peaks were selected, narrowing the max G’ SNPs. The QTL with the highest G’ value (10.33) was present in the first part of chromosome 14 (namely chr. 14a - Table 2); a second region (14b) was spotted in the last part of this chromosome with a max G’ of 8.31. In chromosomes 4 and 12, peaks were detected at 8.18 Mb (4) and 2.11 Mb (12), with a max G’ value of 8.34 and 6.34, respectively (Table 2).

**Table 2:** Statistics on selected QTL for male sterility: details on the peak regions, genes and SNP with their potential impact are reported

										SNP impact			
Chr	sub-region	Start	end	Length	*N*° SNPs	avg. SNPs/Mb	max G’	pos. Max G’	G’ candidate range (bp)	genes	LOW	MODERATE	HIGH
4	-	5,527,229	23,739,762	18,212,533	72,638	3,988	8.34	8,176,221	chr4:7,544,909-8,425,655	60	257	263	14
12	-	1,702,450	3,773,932	2,071,482	3,794	1,832	6.34	2,111,758	chr12:2,040,412-2,945,755	82	55	69	3
14	a	202	9,655,187	9,654,985	34,986	3,624	10.33	444,700	chr14:202–574,964	55	123	108	12
14	b	13,943,952	27,631,802	13,687,850	56,370	4,118	8.31	25,822,452	chr14:25,330,927 − 25,875,057	43	61	41	2

### Candidate genes over QTL regions

*Chromosome 4* - A 18.2 Mb QTL region was identified (5.53–23.74 Mb, Fig. 2b). The G’ peak (8.34, Fig. 2f) was located at 8.17 Mb, and the narrowed region around it was ∼ 800Kb long (7.54-8.43 Mb). Sixty genes were present in the peak region, and 18,417 SNPs were identified between the parents (Suppl. Table S2). Among them, 263 SNPs showed a moderate impact, whilst 14 were categorized as high impact SNPs. A 200Kb interval (8.07 Mb-8.27 Mb) around the peak was further scanned and nine genes were present in this interval (Suppl. Table 2): (i) a Leucine-Rich Repeats (LRR) receptor-like serine/threonine-protein kinase, (ii) the Protein IMPAIRED IN BABA-INDUCED STERILITY 1 (IBS1), involved in female fertility [31], (iii) two isoforms of Formin-like protein 20 (FH20), (iv) the E3 ubiquitin-protein ligase PRT1, and (v) four unknown proteins. No high impact mutations were present in these genes, and the ones with moderate impact and in homozygous state were checked with Provean without predicting any deleterious effects (data not shown).

*Chromosome 12* - A 2.07 Mb QTL region was highlighted (1.70-3.77 Mb, Fig. 2c). The G’ peak (6.34) was located at 2.11 Mb (Fig. 2g), and the narrowed region around it was ∼ 800Kb long (2.04–2.90 Mb). Seventy-nine genes were annotated in this region, as well as 10,740 SNPs (Suppl. Table S2). Among them, 68 SNPs showed a moderate impact, whilst three were categorized as high impact SNPs. A 100 kb interval (2.05–2.14 Mb) around the peak was scanned and six genes were identified: (i) an aldehyde oxidase (GLOX1), (ii) a mitochondrial outer membrane protein porin of 36 kDa, (iii) a B3 domain-containing protein, (iv) a pentatricopeptide repeat-containing protein (PCMP-E22), belonging to a class of genes reported as involved in cytoplasmatic male sterility restoration in rice and *Arabidopsis* [32, 33], (v) a LRR receptor-like serine/threonine-protein kinase, and (vi) a MYB124 transcription factor. No high impact mutations were present in these genes, and the ones with moderate impact and in homozygous state were checked with Provean without predicting any deleterious effects (data not shown).

**Fig. 3:**
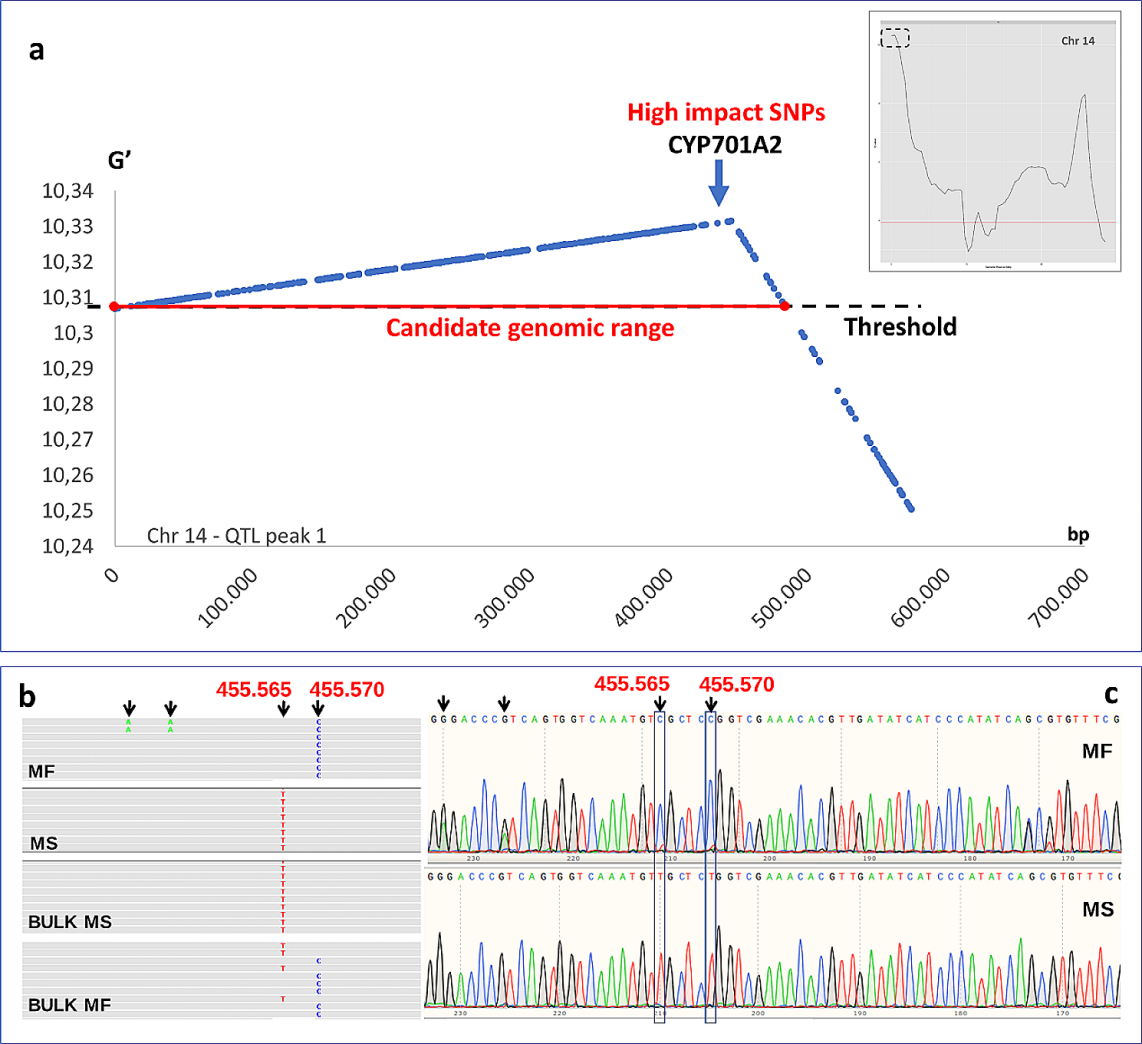
**a**) Detail of the 14a, one of the two G’ peaks in chromosome 14; **b**) Alignment profile with Illumina based reads of the two parents (MF and MF) and the two bulks (MS and MF) in the peak region. **c**) Sanger validation of the SNPs observed with Illumina sequencing

*Chromosome 14 -* Two G’ peaks were spotted (named 14a and 14b, Fig. 2d and e, respectively). The first one included a 9.45 Mb long region (0.2–9.65 Mb, Fig. 3) with a G’ peak (10.33) at 0.44 Mb (Fig. 2h). By focusing on a region of ∼ 500 kb (0-0.57 Mb) around the peak, fifty-five genes and 7,818 SNPs were identified between the parents (Suppl. Table S2), including a 3-ketoacyl-CoA synthase-like gene, a class of proteins that have been reported to be involved in the pathways of cuticular wax and cutin biosynthesis [34]. Among them, 108 SNPs showed a moderate impact, whilst 12 were categorized as high impact SNPs. This region was further investigated within a ∼100 kb interval (0.43–0.54 Mb), highlighting three high impact mutations, together with several moderate impact variants. Overall, twelve genes were identified, including (i) a pentatricopeptide repeat-containing protein, a class of genes indicated has players in pollen development and cytoplasmatic male sterility restoration in rice and *Arabidopsis* [32, 33], (ii) a calcium-transporting ATPase (LCA1), (iii) a serine/arginine-rich splicing factor 31 (RS31), (iv) a bifunctional fucokinase/fucose pyrophosphorylase (FKGP), (v) an aluminum-activated malate transporter (ALMT10), (vi) a cytochrome P450 (CYP703A2), belonging to the CYP703 family, reported to provide sporopollenin building blocks during pollen development [35], and (vii) a histone-lysine N-methyltransferase (ATX4). Three high impact mutations were detected in three different genes with unknown function, together with some moderate impact SNPs in homozygous state. All the missense variants were checked with Provean and none, except two, were predicted to have deleterious effects. The first was in the ATX4 gene (104 kb far from the G’ peak), while the second was highly closed to the G’ peak (10 kb apart). The latter (455,565 bp) was a missense variant (G1271A) present in the CYP703A2 gene (V2_14g000490.1), a cytochrome P450 involved in the exin synthesis (Suppl. Table 2). The mutation was predicted to produce an amino-acid substitution (Arg424Gln), which Provean analysis reported as highly deleterious (score − 6.392).

A second peak (14b) surrounded a 13.69 Mb long QTL region (13.94-27.63 Mb, Fig. 2e) with a G’ peak (8.31) at 25.82 Mb (Fig. 2i). By including a region of ∼ 500 Kb (25.3–25.9 Mb) around the peak, 43 genes and 5,650 SNPs were highlighted between parents (Suppl. Table S2). Among them, 41 SNPs showed a moderate impact, whilst 2 were categorized as high impact SNPs. By focusing on a 100 kb region around the peak (14b; 25.72–25.92 Mb), two genes were identified. Both genes coded for unknown proteins, showing one SNPs in heterozygous state, with a low/moderate impact.

### Derived cleaved amplified polymorphic sequences (dCAPs) marker design

**Fig. 4:**
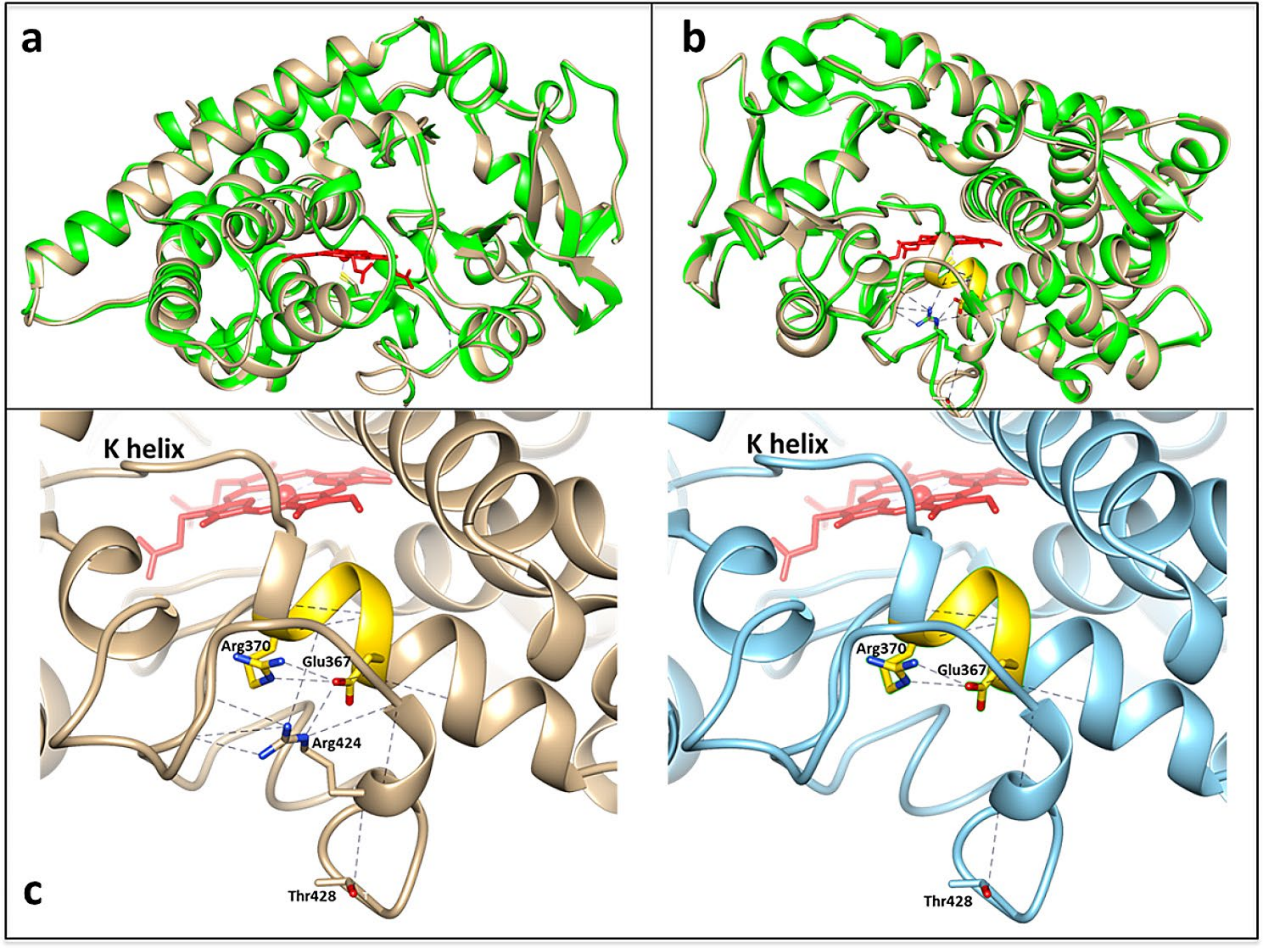
**a**) CYP76AH1 crystal structure from *Salvia miltiorrhiza* used for homology modeling of CYP703A2; **b**) Modeled globe artichoke WT CYP703A2 protein structure; **c**) Comparison between the models of CYP703A2 protein in globe artichoke MF (left) and MS (right) in the region of interest. ERR triad is highlighted in yellow on K helix, the amino acids interacting with the mutated site are shown, as well as the heme (in red)

**Fig. 5:**
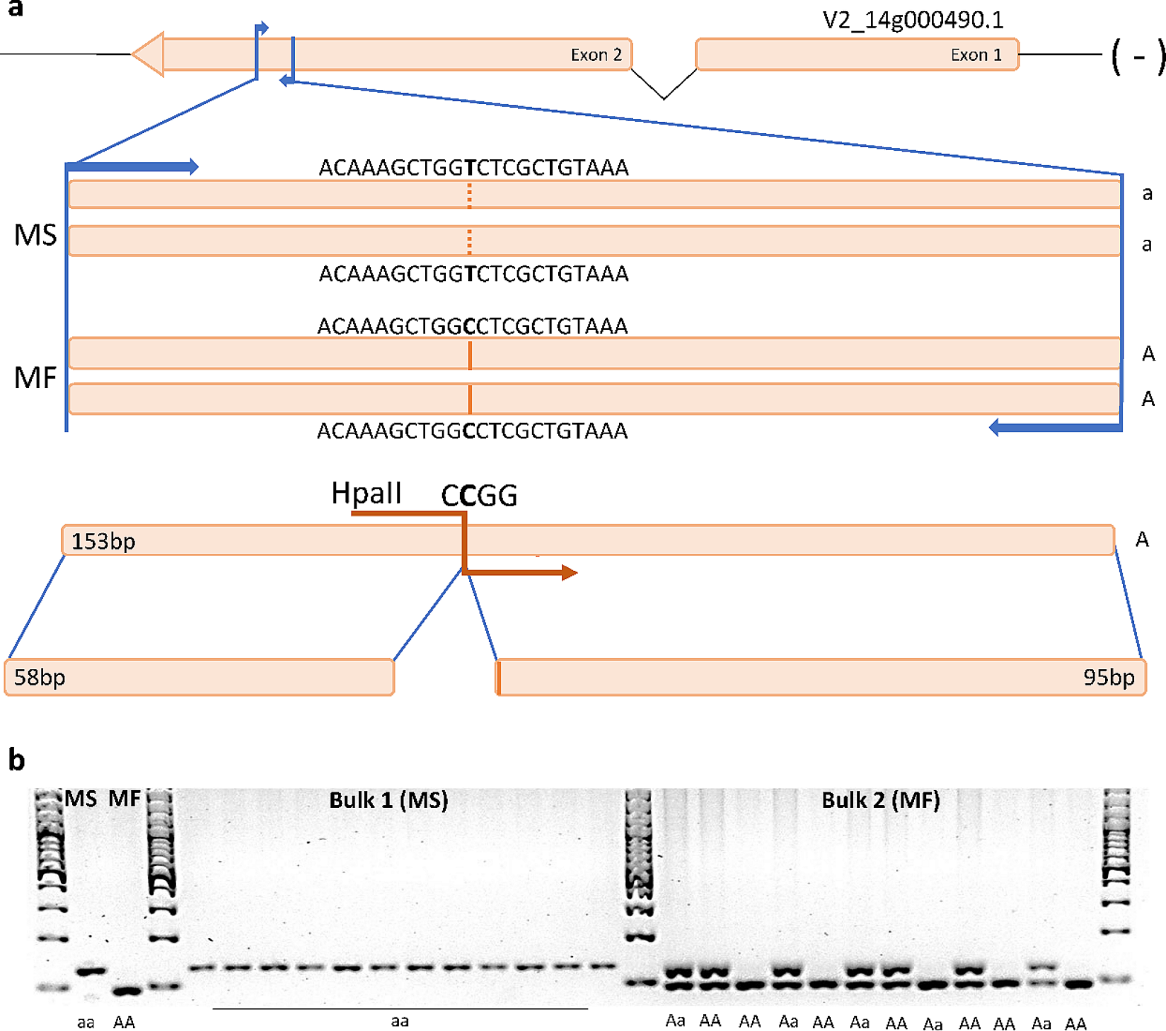
**a**) *V2_14g000490.1* (*CYP703A2*) gene structure. The two alleles are reported, as well as the sequence sizing produced by enzymatic cut with HpaII; **b**) Validation of the haplotypic designed CAPs marker (14-455570-ms) in the population. From left to right, parental lines (MS and MF) are shown, followed by Bulk 1 (one uncut fragment) and Bulk 2 (both heterozygous and recessive homozygous states are present)

To allow the discrimination between MS and MF genotypes within the F_2_ population, a single dCAPs marker was developed around the SNP located at 455,565 bp in chromosome 14. As this SNP was not associated with any restriction enzyme, a neighbor one (455,570 bp), belonging to the same male sterile haplotype, was selected for the development of a marker for the locus, as part of the HpaII (CCGG) restriction site (Fig. 4). The developed marker (named *14-455570-ms*) was validated on the F_2_ population, demonstrating to be useful in the assessment of the MS/MF phenotype (Supp. Figure 1 and Supp. Table 3). As depicted in Fig. 3, the non-reference allele (AA) identified in the MF parent allowed the restriction cut of the amplified region in two co-migrating sequences of 95 bp and 58 bp. The cut (MF - AA) and uncut (MS - aa) sequences can be easily visualized by gel electrophoresis (Fig. 5), and their segregation in the population allowed the recognition of the Bulk 1 from the Bulk 2. As expected, “aa” genotypes were only present in the MS bulk (Bulk 1), while dominant homozygous (AA) and heterozygous (Aa) states were present in the MF bulk (Bulk 2). The validation of the marker on the whole F_2_ population confirmed a 3:1 (MF: MS) segregation of the locus, strongly associating the dCAPs marker (*14-455570-ms*) with the observed phenotypic segregation.

### Sequence validation and Arg424Gln investigation through CYP703A2 homology modeling

The genomic region containing the Arg424Gln missense variant in CYP703A2 was inspected in the MS and MF parental lines and it was found fixed in homozygous state in the MS parent (Fig. 3b and c). Such mutations were further validated through Sanger sequencing of the region (Fig. 3c). According to both Sanger and Illumina-based resequencing alignment (Fig. 3b), a cytosine in position 455,565 (reference-like) in the MF parent, was mutated in a thymine in the MS parent (Suppl. Table 2), leading to the substitution of Arg424 with a Gln (Suppl. Table 2). The mutation on CYP703A2 was investigated through protein modeling, to assess the effective impact of the predicted amino acid substitution (Arg424Gln) on protein structure. A homology model was built (see Experimental Procedures) using the best matching crystal structure, CYP76AH1 from *Salvia miltiorrhiza*, sharing 31% of primary sequence identity and 50% of homology, as template (Fig. 3a and b). The analysis highlighted the role of Arg424 as the amino acid involved in a salt bridge with a glutamate residue that is part of the fingerprint motif EX1 × 2R (Fig. 4a), located on helix K. Together with the E and the R from this motif, Arg424 is part of the so-called ERR triad, a conserved region in the superfamily involved in salt bridges and protein folding [36].

## Discussion

### Male sterility in globe artichoke

Even if globe artichoke is traditionally vegetatively propagated, this form of propagation has a great impact on production costs, which tend to be higher for the intensive labor required for transplanting and plant losses in field preparation. On the other hand, the use of the achene as a reproductive unit makes it possible to treat the crop as annual, increasing field uniformity and reducing planting costs, as well as pathogens diffusion [37]. In the last decades, this has pushed the popularity of seed-propagated cultivars and the production of F_1_ hybrids which, in some cases through the exploitation of male sterile (MS) genotypes, have been successfully introduced in cultivation [11]. Indeed, to take advantage of the low seed costs and the heterosis phenomenon through hybrid production, it is essential to have an effective globe artichoke male-sterility system available [16], which allows to avoid selfing and easies the crossing process. Several genotypes are today available carrying MS phenotype [11], but the genetic bases of this trait have been, to date, poorly explored.

### Investigating globe artichoke genetics with BSA-seq, allowing marker development for breeding

We analyzed an F_2_ population of 250 offsprings derived from a cross of a MS globe artichoke with a male fertile (MF) cultivated cardoon *(C. cardunculus* var. *altilis*), segregating to produce vital/not vital pollen, and fitting a monogenic Mendelian 3:1 model. This population was here analyzed with the BSA-seq approach [38], previously proven to be efficient for monogenic/oligogenic character spotting and QTL mapping in many different species [39–47]. Such technology was applied to fully address the genetic determinant of MS and overcome limitations due to the lower resolution power of SRAP markers, previously highlighted by Zayas et al [16]. BSA-seq analysis revealed four chromosomal regions (4, 12, 14a and 14b) putatively involved in male sterility (Fig. 2). A QTL with the noticeably highest G’ value (10.33) was detected in the first part of chromosome 14. In the paper by Zayas et al [16] five candidate regions were already spotted as a candidate for the MS trait, and one revealed to be present on chromosome 14, in a proximal chromosomal region. By focusing on the 14a region (Fig. 3a), in the 20 kb around the peak position (444,700 bp), four genes were spotted, as well as 5 SNPs, of which only one was predicted to produce a homozygous missense variant. The *14-455570-ms* marker validation, conducted on the whole F_2_ population, confirmed its strong association with the observed male sterility trait (Supp. Figure 1). The male sterile phenotype was always observed in the population in association with a single recessive mutation in the second exon of gene, while wild type and heterozygous individuals in the progeny had a fertile phenotype. This is consistent with the results in rice [48] and Arabidopsis [35], where phenotypes are alternatively caused by a single recessive mutation in the CYP703A2 gene, causing a coding frame shifting in the second exon, and a homozygous T-DNA insertion in the gene, respectively.

### Investigating a candidate gene for male sterility

The mutated gene was a cytochrome P450 (CYP703A2, V2_14g000490.1) coding for a protein responsible for sporopollenin synthesis [35]. Many genes are known to be involved in the sporopollenin synthesis/transport [49], and plants with mutations in these genes have severe defects in exin/sexine layers, anther cuticle, and are usually complete male sterile [50]. As example, CYP703A3 belongs to the CYP71 clan, which catalyzes the biochemical pathway of fatty acid hydroxylation [51]. It has been demonstrated that the same cytochrome P450 hydroxylase, in presence of NADPH, is able to catalyze *in-chain* hydroxylation of FAs as sporopollenin precursors, with a preferential hydroxylation of lauric acid at the C-7 position [35]. This gene has also been reported to be essential for the development of anther cuticle and pollen exine in rice, where mutants showed a fully pollen sterile phenotype [48]. In Arabidopsis, CYP703A2 mutants produce partially sterile pollen grains displaying abnormal exine and sporopollenin deposition [35]. The crucial role of this enzyme in reproductive tissues has been also shown by its overexpression in *Arabidopsis*, where altered expression through transgenesis was able to increase silique size and seed number, altering the contents of fatty acids composition of cutin monomer in the siliques [51]. In our case, the mutation in the globe artichoke CYP703A2 generated an amino-acid substitution (Arg424Gln) with a highly predicted deleterious effect. We investigated this effect through a 3D analysis of the protein (Fig. 4), confirming Arg424 as the amino acid involved in a salt bridge with a glutamate residue within the key motif EX1 × 2R (ERR) in helix K. The ERR triad is highly conserved across the cytochrome P450 superfamily as it is involved in salt bridges, essentials for a correct folding and heme incorporation [36, 52, 53]. It has been widely reported that mutations of the residues of the ERR triad in different cytochromes P450 resulted in loss of function due to the lack of heme incorporation [54–59]. Here, Gln424 in the mutated protein likely interferes with the salt bridge formation with Glu367 in helix K, potentially leading to the loss of the ERR triad (Fig. 4c). The convergence over this gene in different species (both monocot and dicots) is suffragated by the crucial role of this specific p450 in the sporopollenin synthesis [35, 49], coupled with the presence of a single copy of the gene in all the investigated plant species [35, 50, 60]. Accordingly, genomic analysis of globe artichoke confirmed the presence of the gene in a single copy. Despite the absence of established protocols for gene knockout in globe artichoke, this enzyme presents itself as a prime target for CRISPR/Cas9 genetic manipulations. However, considering the male sterility observed upon loss of function in CYP703A2 in both monocot and dicot plants [35, 50], together with the extensive list of literature in yeast highlighting the deleterious effects of mutations in the conserved ERR triad [36, 52, 53], it is reasonable to deduce that a similar knockout may yield little additional insight.

### Breeding perspectives for male sterility

The discovery and markers design for the locus on top of chromosome 14, and its association with male sterility in globe artichoke undoubtedly opens new breeding possibilities, bringing to light the potential for leveraging male sterility to enhance hybrid seed production. However, the route from genetic discovery to practical application in breeding programs is highly challenging. The complexity of male sterility, influenced by genetic and environmental factors, might benefit from the integration of traditional approached with modern computational strategies, such as machine learning, to successfully integrate this trait in breeding programs. By investigating syntenic loci from model and well-studied species, minor crops can benefit from a mole of research material that could be hardly achievable to produce [61]. Moreover, harnessing ML algorithms to predict male sterility genomic architectures, researchers can gain insights into the underlying genetic interactions and environmental dependencies. This predictive capability can extend beyond traditional breeding approaches, allowing for the anticipation of breeding outcomes in diverse environmental contexts. The use of functional genomics serves as a foundation for these predictive models [62, 63], offering a deeper understanding of gene function and regulation in the context of male sterility. Moreover, the application of predictive breeding promises to enhance the precision and efficiency of breeding programs [64–67]. By leveraging the genomic bases of complex polygenic adaptive trait architectures, predictive molecular breeding can potentially overcome the challenges posed by traits like male sterility, which may exhibit Mendelian inheritance patterns yet are influenced by a multitude of factors. The exploration of these novel perspectives, can unlock new possibilities for the development of superior hybrids, thereby contributing to sustainable agricultural practices and food security.

## Conclusions

The present research on globe artichoke male sterility highlights the potential of using genetic markers, such as the developed dCAPS marker, for breeding programs aimed at enhancing crop yields through hybrid seed production. By elucidating a genetic basis for male sterility, this study provides a template for further research in other plant species, potentially leading to breakthroughs in agricultural productivity and sustainability. Moreover, by enabling the development of more efficient and cost-effective plant breeding, the broader implications of these findings underscore the importance of genetic research in addressing global food security challenges.

### Electronic supplementary material

Below is the link to the electronic supplementary material.

Supplementary Material 1

Supplementary Material 2

Supplementary Material 3

Supplementary Material 4

## Data Availability

Sequencing data used in this study are openly available in the NCBI database (PRJNA892759).
