# Supplementary figures and images for: The Dark Side of the pollen: BSA-seq identified genomic regions linked to male sterility in globe artichoke
Source: BMC Plant Biol. 2024 May 17;24:415. doi: 10.1186/s12870-024-05119-z (PMC11100218; doi:10.1186/s12870-024-05119-z)

**Supplementary Figure 1** - HpaII dCAPS genotyping agarose gel for the whole F2 population.


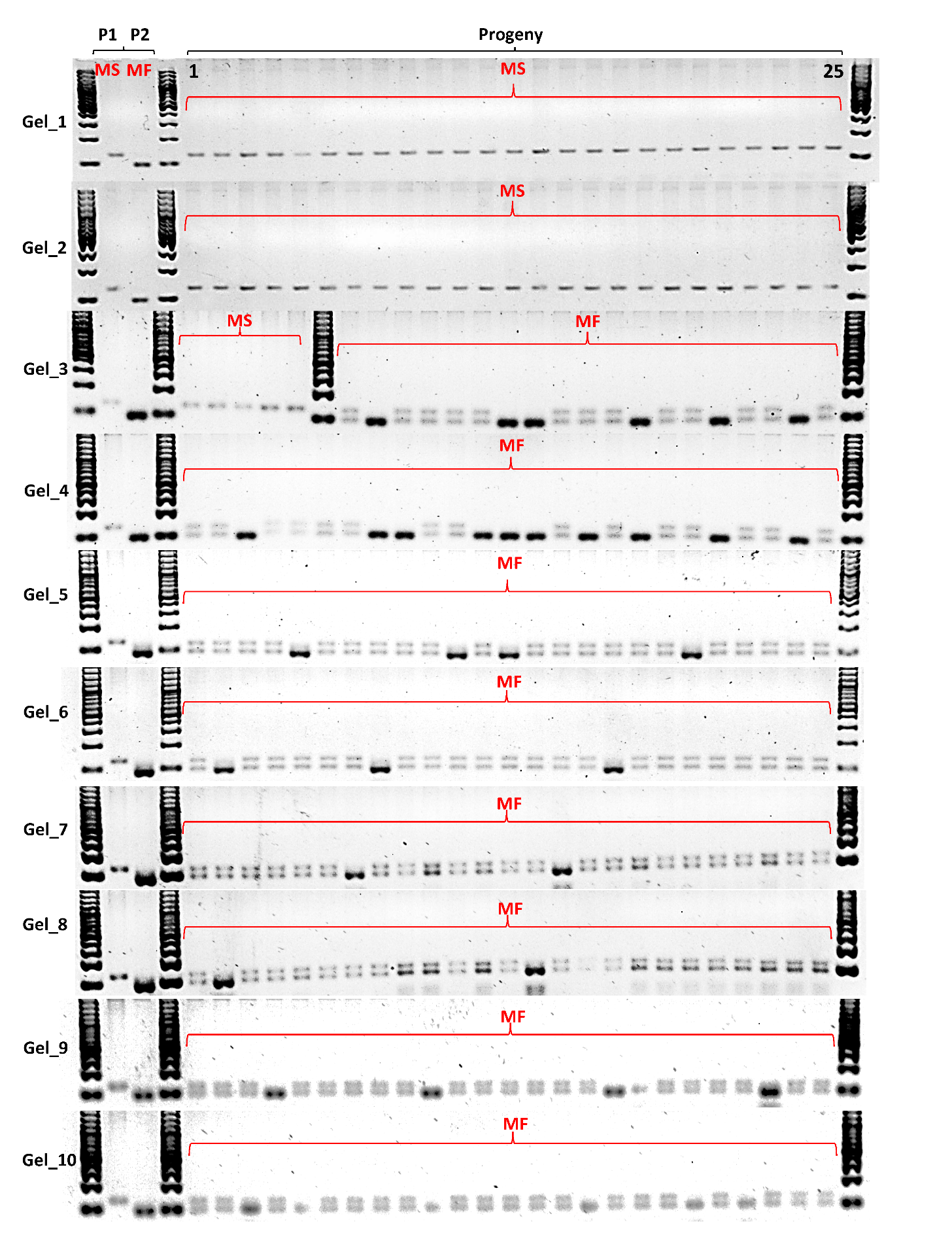

Supplement: Supplementary file 3 — Supplementary Material 3 [file 12870_2024_5119_MOESM3_ESM.docx]
